# Fertilization measures improve agronomic traits of alfalfa by regulating root bacterial co-occurrence networks

**DOI:** 10.3389/fpls.2026.1882294

**Published:** 2026-07-29

**Authors:** Wentao Mi, Shuang Liang, Ru Meng, Weibo Ren, Ting Yuan, Min Wang, Xiaona Zhang, Hao Zheng, Feng Yuan, Chengchen Pan

**Affiliations:** 1 Key Laboratory of Ecology and Resource Use of the Mongolian Plateau, Ministry of Education, Hohhot, China; 2 Inner Mongolia Key Laboratory of Grassland Ecology, School of Ecology and Environment, Inner Mongolia University, Hohhot, China; 3 Office of Scientific Research, Inner Mongolia University of Finance and Economics, Hohhot, China; 4 Department of Architectural Engineering, Taishan Polytechnic, Tai’an, Shandong, China; 5 Inner Mongolia Institute of Forestry and Grassland Monitoring and Planning, Hohhot, China; 6 National Center of Pratacultural Technology Innovation, Hohhot, China; 7 Northwest Institute of Eco-Environment and Resources, Chinese Academy of Sciences, Lanzhou, Gansu, China

**Keywords:** agronomic traits, co-occurrence network, fertilization, rhizosphere bacteria, soil nutrients

## Abstract

**Introduction:**

Alfalfa (*Medicago sativa*; L., Fabaceae) is an important leguminous forage crop, and rational fertilization is critical for improving its productivity; however, the synergistic mechanisms by which fertilization regulates plant–soil–microorganism interactions through rhizosphere microbial networks remain poorly understood.

**Methods:**

We examined the effects of 14 fertilization treatments—including a nonfertilized control, single-nutrient-deficient treatments, and treatments with varying or imbalanced N–P–K ratios—on soil properties, agronomic traits, and rhizosphere bacteria in alfalfa based on a two-year “3414” fertilization experiment.

**Results:**

All fertilization treatments significantly (p < 0.05) increased soil nutrient content, and, due to the interaction between the cumulative effects of fertilizers and associated biological processes, soil nutrient levels differed significantly between years. At the same time, fertilization significantly improved alfalfa agronomic traits (e.g., height, stem diameter, and yield), with the optimal fertilization treatment (N2P2K2: 135 kg ha^-1^ urea, 175 kg ha^-1^ calcium superphosphate, and 120 kg ha^-1^ potassium sulfate) showing the greatest improvement. Microbial analysis revealed a lag in the response of bacterial diversity to fertilization, leading to significant interannual changes. Microbial analysis revealed that bacterial diversity varied significantly between years, while its response to fertilization was generally consistent across the two years. The co-occurrence network analysis showed that although node number did not differ significantly among treatments, the rhizosphere bacterial network under optimal fertilization (N2P2K2) exhibited markedly fewer edges and lower mean degree, density, and clustering coefficient than under the nutrient-deficient or NPK-ratio-imbalanced treatments, reflecting a “simple and efficient” topology with fewer redundant microbial interactions. In contrast, the latter treatments exhibited a “complex redundancy” pattern characterized by denser, more highly interconnected networks. Path analysis further confirmed that fertilization could improve alfalfa agronomic traits through direct and indirect pathways.

**Conclusion:**

This study demonstrates that optimal NPK fertilization enhances alfalfa productivity by simplifying and stabilizing the rhizosphere bacterial co-occurrence network, providing a microbiological basis for precision fertilization management in leguminous forage production.

## 1 Introduction

Intensive farming is paramount in the modern economy. On one hand, it provides a rich source of food for human beings; on the other hand, it also makes a substantial contribution to the development of the national and local economy (Henchion et al., 2017, 2021; Petersen and Snapp, 2015; Pulina et al., 2018). This breeding model has advanced the circular economy by effectively integrating resources across industries. In particular, integrating breeding and planting has greatly reduced the carbon footprint of the breeding industry, achieved ecological balance, and promoted the development of a green economy. However, with the rapid development of intensive farming, ensuring a sufficient, low-cost, high-nutrient forage supply has become a crucial part of this approach (Rudel et al., 2015; Kulkarni et al., 2018).

Alfalfa (*Medicago sativa*; L., Fabaceae) is particularly suitable for large-scale cultivation due to its strong adaptability, high nutritional quality, and short growth cycle (Gu et al., 2018; Wei et al., 2025). This crop has become an important forage resource and plays a crucial role in animal husbandry. At present, more than 80 countries worldwide are planting alfalfa, covering a total area of more than 30 million ha, making it one of the most widely used forage types worldwide (Chen et al., 2020; Zhang and Wang, 2025). At the same time, alfalfa has strong resistance to grazing and mowing, enabling one-time input and long-term utilization, thereby markedly improving its economic value in feed production (Mielmann, 2013; Singer et al., 2018). Additionally, alfalfa has nitrogen-fixation ability and soil and water conservation functions, and is widely used in ecological and agricultural fields, such as the restoration of degraded grasslands and the construction of artificial grasslands (Weidner et al., 2003; Jiang et al., 2007; Yu and Zhu, 2025). However, several challenges remain in achieving a stable and efficient supply of alfalfa in intensive farming systems. The most important of which is how to synergistically increase its yield and reduce production costs through scientific field management (such as precision fertilization).

Soil moisture and nutrients are the basis of plant growth and development (Oldroyd and Leyser, 2020). In agricultural production, nutrient limitations are typically the primary cause of slow plant growth and low yields (Jia et al., 2022; Singh et al., 2024). Therefore, fertilization has become an important measure for improving crop yield (dos Santos et al., 2018; Barlog et al., 2022). Reasonable fertilization can rapidly increase soil nutrient content and alleviate nutrient limitations during plant growth, thereby promoting rapid growth and increasing yield. However, excessive fertilizer use may have many negative effects, including long-term soil acidification or salinization, resulting in nutrient imbalances and soil compaction (Barabasz et al., 2002). Long-term excessive fertilization will also lead to decreased diversity of the soil microbial community and a single compositional structure, affecting the stability of soil ecological functions (Xu et al., 2020; Zhang et al., 2022). In addition to affecting soil and microorganisms, an imbalance in fertilizer ratios considerably exacerbates the limiting effects of nutrient deficiencies, further inhibiting plant growth and yield (Marques Teixeira et al., 2021; Liu et al., 2024). Therefore, only the scientific and rational use of fertilizers can ensure the sustainable development of soil health and the agricultural environment while simultaneously increasing crop yields (Chen et al., 2014; Geisseler and Scow, 2014; Betancur-Corredor et al., 2023). The “3414” experiment is a commonly used method for combining fertilizer treatments. By mixing nitrogen (N), phosphorus (P), and potassium (K) fertilizers across four different gradients, only 14 treatment combinations can be used to comprehensively study the effects of these three major fertilizer elements in the field and to examine their synergistic effects to the greatest extent. This method offers convenience and space-saving advantages and provides an effective experimental design for fertilizer research (Chen et al., 2019; Duan et al., 2023).

The rhizosphere is a key interface for plant–soil–microorganism interactions and plays an important role in nutrient acquisition and biogeochemical cycling (Ryan et al., 2009). Plant roots release photosynthetically fixed carbon into the surrounding soil through root exudates, thereby providing substrates for rhizosphere microorganisms and shaping distinct microbial communities (Jones et al., 2004a; Badri and Vivanco, 2009). These rhizosphere microorganisms are closely involved in nutrient transformation, root development, plant growth, and stress adaptation, suggesting that the rhizosphere microbiome is an important biological component of sustainable crop production (Berendsen et al., 2012; Zhang et al., 2017). However, microbial communities are not simply collections of individual taxa; rather, they are organized through complex potential interactions. Co-occurrence network analysis provides a useful approach for exploring these potential relationships among microbial taxa and for evaluating community organization at the network level. Network topological parameters, such as nodes, edges, average degree, and modularity, can help characterize the complexity and potential stability of microbial communities (Ma et al., 2020; Mi et al., 2026). Therefore, analyzing rhizosphere bacterial co-occurrence networks may provide deeper insight into how different fertilization regimes affect microbial community organization and how these changes are associated with alfalfa agronomic performance.

Previous studies on fertilization in alfalfa have mainly focused on yield, forage quality, soil nutrient status, or microbial community composition. These studies have improved our understanding of how fertilization affects alfalfa production and soil microbial communities. However, most of them have examined plant traits, soil properties, or microorganisms separately, and the relationships among these components under different fertilization regimes remain insufficiently understood. In particular, limited attention has been paid to whether changes in the rhizosphere bacterial co-occurrence network are associated with changes in alfalfa agronomic traits. Therefore, this study aimed to investigate the effects of different fertilization treatments on alfalfa agronomic traits, rhizosphere bacterial community composition, and bacterial co-occurrence network structure. Specifically, we addressed two questions: (1) how different fertilization treatments affect the composition and co-occurrence network structure of rhizosphere bacterial communities, and (2) whether changes in alfalfa agronomic traits are associated with shifts in the topological parameters of rhizosphere bacterial co-occurrence networks.

## 2 Materials and methods

### 2.1 Experimental design

This study was conducted in Hohhot, Inner Mongolia Autonomous Region, China (coordinates: 111° 46’ 57.079” E, 40° 54’ 3.402” N), based on a “3414” experimental design. The “3” in the experiment represented three fertilizers: nitrogen fertilizer (urea, N ≥ 46%), phosphate fertilizer (calcium superphosphate, P_2_O_5_ ≥ 61%), and potassium fertilizer (K_2_SO_4_ ≥ 52%). The number “4” represents the four fertilization levels set in this experiment. Specifically, level 0 means no fertilization, and levels 1, 2, and 3 are 0.5, 1.0, and 1.5 times the local optimum fertilization amount, respectively. The local optimum fertilization amounts (level 2) were 135 kg ha^-1^ urea, 175 kg ha^-1^ calcium superphosphate, and 120 kg ha^-1^ potassium sulfate (Wu, 2007). The number “14” represents the 14 treatment combinations included in this study. The specific N–P–K application rates and treatment background for each treatment are detailed in **Table 1**.

**Table 1:** Fertilization treatments and fertilizer application rates used in this study.

Treatment	Abbreviation	Fertilizer application amount (kg·ha^−1^)			Treatment description
		Urea	Calcium superphosphate	Potassium Sulfate	
N0P0K0	CK	0.00	0.00	0.00	Control
N0P2K2	N-def	0.00	175.00	120.00	Nitrogen-deficient treatment
N1P2K2	Low-N	67.50	175.00	120.00	Low-nitrogen treatment
N2P0K2	P-def	135.00	0.00	120.00	Phosphorus-deficient treatment
N2P1K2	Low-P	135.00	87.50	120.00	Low-phosphorus treatment
N2P2K2	Opt-F	135.00	175.00	120.00	Optimal fertilization treatment
N2P3K2	High-P	135.00	262.50	120.00	High- phosphorus treatment
N2P2K0	K-def	135.00	175.00	0.00	Potassium-deficient treatment
N2P2K1	Low-K	135.00	175.00	60.00	Low-potassium treatment
N2P2K3	High-K	135.00	175.00	180.00	High-potassium treatment
N3P2K2	High-N	202.50	175.00	120.00	High-nitrogen treatment
N1P1K2	Low-NP	67.50	87.50	120.00	Low-nitrogen and -phosphorus treatment
N1P2K1	Low-NK	67.50	175.00	60.00	Low-nitrogen and -potassium treatment
N2P1K1	Low-PK	135.00	87.50	60.00	Low-phosphorus and -potassium treatment

**Figure 1:**
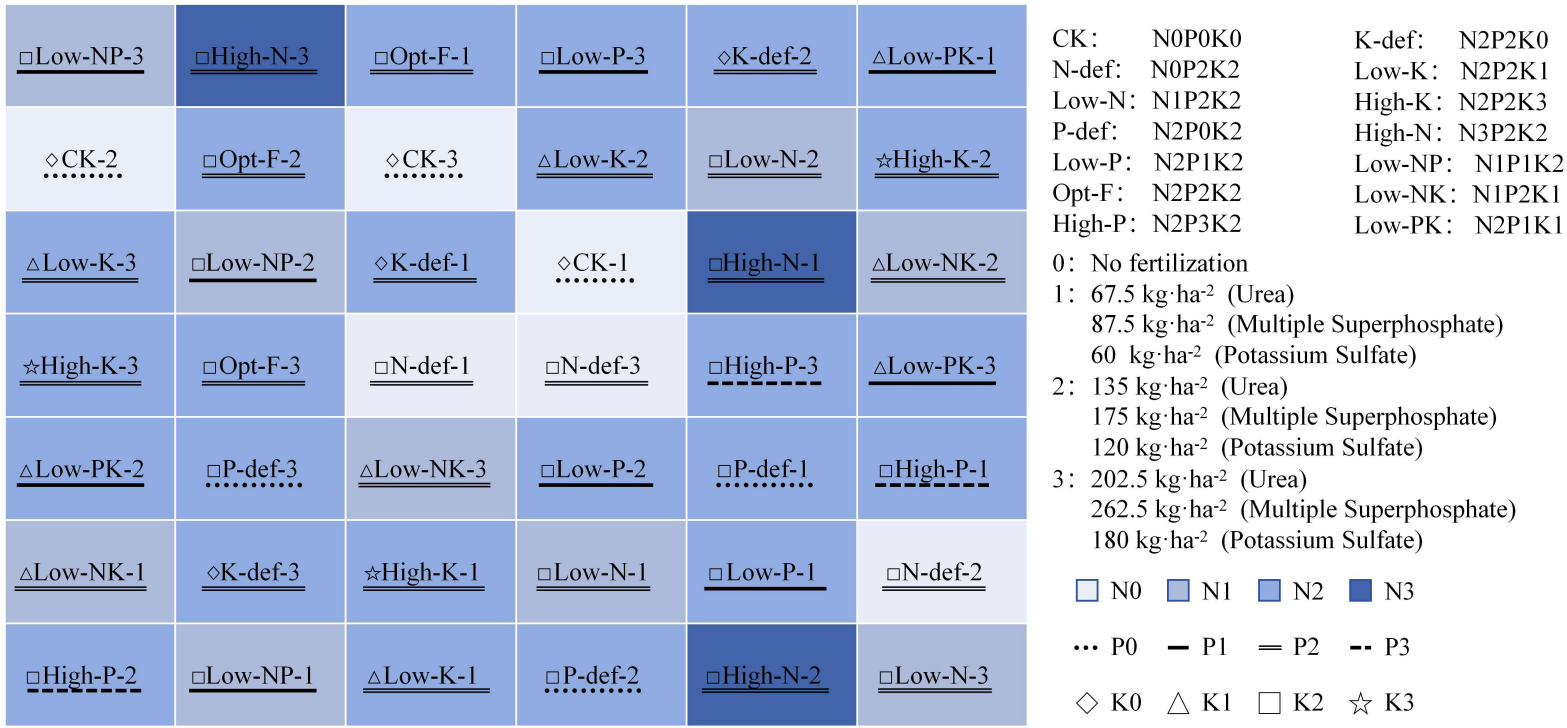
Experimental fertilization treatment gradient and experimental plot distribution map.

The alfalfa variety “WL343” was selected because it is a high-yielding cultivar well adapted to local growing conditions and is among the preferred varieties for large-scale forage production companies in this region. The plants were planted in July 2022, with a sowing depth of 1–2 cm, a row spacing of 60 cm, and each experimental plot measuring 5 m × 3 m (15 m²). In the first year (2022), all plots received the same no-fertilization management, so no treatment-related differences were introduced before the start of the fertilization experiment in 2023. Furthermore, all treatments were randomly assigned to plots, ensuring that any spatial heterogeneity in background soil conditions was randomly distributed across treatments rather than systematically biasing any particular treatment. Fertilization treatments was applied in 2023 and 2024. Three replicate plots were set up for each treatment, for a total of 42 plots. The experimental plots were randomly distributed in the plot (**Figure 1**). A 1-m interval between plots was maintained to prevent nutrient infiltration between plots from affecting the experimental results. The remaining experimental measures were consistent with normal field management.

### 2.2 Agronomic trait measurement

Agronomic traits were measured in mid-August 2023 and 2024. The indicators and measurement methods were as follows:

Plant height and stem diameter: One 1-m^2^ quadrat was randomly set up in each experimental plot. A total of 10 alfalfa plants with good growth and no pests were randomly selected from each quadrat to measure the height from the ground to the main branch growth point. The results were expressed as a mean value, accurate to 0.1 cm. At the same time, the diameter at 1 cm from the base of each alfalfa plant was measured in mm, with an accuracy of 0.1 mm.

Hay yield: One 1-m^2^ quadrats were mowed, and the stubble height was about 5 cm. The harvested alfalfa plants were dried in an oven to a constant weight and converted to yield.

### 2.3 Soil sampling, physical, and chemical properties measurement

After each plot was cut, three alfalfa plants were randomly selected from each quadrat. After carefully removing the non-rhizosphere soil attached to the roots, a disposable brush was used to collect the rhizosphere soil. The rhizosphere soil collected from all plants in the same plot was mixed evenly. One mixed rhizosphere soil sample was obtained from each plot, placed in a sterile centrifuge tube, frozen in liquid nitrogen, and then transferred to an ultra-low-temperature refrigerator at −80 °C for subsequent microbial sequencing. Because the amount of rhizosphere soil obtained from the alfalfa root system per plant is small, and this study involves determining many soil physical and chemical indicators, using rhizosphere soil directly for analysis may affect subsequent research on this experimental platform. Therefore, nonrhizosphere soil was used to determine soil nutrients. The nonrhizosphere soil was collected from the adjacent position to the rhizosphere soil sampling point and stored at 4 °C for analysis of soil physical and chemical properties. The soil physical and chemical determination methods were shown in **Supplementary Table 1**.

### 2.4 Microbial sequencing

The soil microbial community structure was analyzed using high-throughput 16S rDNA sequencing. Genomic DNA was extracted from rhizosphere soil samples using a HiPure Stool DNA kit (Magen, Guangzhou, China) according to the manufacturer’s protocol. Bacterial primers were 341F: CCTACGGGNGGCWGCAG and 806R: GGACTACHVGGGTATCTAAT. The HiPure Stool DNA kit was used to amplify the V3–V4 region (Yuan et al., 2023). DNA library sequencing was performed on Illumina Novaseq 6000. The raw high-throughput sequencing data for bacteria were deposited in the NCBI Sequence Read Archive (SRA) under the accession PRJNA1438644. Subsequently, the community composition and diversity indices of the rhizosphere bacterial community were characterized.

### 2.5 Statistical analysis

R (V4.5.2) was used for the normality test and the homogeneity of variance test. Multiple comparisons were performed using the Tukey Honestly Significant Difference test at the 0.05 significance level. The *linkET* and patchwork packages in R were used to perform a Mantel test on microbial and environmental data. A Mantel test was conducted to assess whether the variations in soil physicochemical properties under different fertilization treatments were significantly correlated with shifts in microbial community structure, thereby elucidating the role of soil environmental variables in shaping the rhizosphere bacterial community. For co-occurrence network analysis, samples from the same fertilization treatment in 2023 and 2024 were pooled before network construction. This was because each treatment contained only three biological replicates in a single year, which was insufficient for stable correlation-based network inference. Pooling the two years increased the number of samples per treatment to six and improved the reliability of network topological parameters. This study applied multiple testing corrections using the Benjamini–Hochberg method to control the false discovery rate. We calculated the Spearman correlation coefficient at the genus level for each pair of bacteria. We retained only those with an absolute correlation coefficient greater than 0.8 and a p-value less than 0.05 (Fan et al., 2021). The analysis aims to retain only highly trusted co-occurrence relationships and reduce false associations arising from weak correlations, thereby reliably identifying the dominant species in the network. The nodes in the network represent genus-level bacteria, and the edges represent a significant correlation between them. Additionally, this study constructed a subnetwork of the co-occurrence network for each treatment to identify significant differences in topological parameters across treatments (Liu et al., 2025). *igraph* was used to compute the topological parameters of the co-occurrence network (Csardi and Nepusz, 2006). This study examined nine topological parameters of co-occurrence networks, including modularity, the number of positive and negative correlations, the number of nodes and edges, the average clustering coefficient, the average degree, the centralized degree, and graph density.

We used the *plspm* (Sanchez et al., 2013) package in R to construct a partial least squares path model (PLS-PM) to elucidate the comprehensive mechanism by which fertilization influences the “plant–soil–microorganism” system. The initial step in model construction was Z-standardizing agronomic traits, soil, and microbial data to eliminate dimensional differences among them. This procedure ensures that variables can be compared on a common scale, thereby avoiding the disproportionate impact of a single variable on the model due to differences in units. We then defined the latent variable and its corresponding measurement variable in the PLS-PM function. A 0–1 matrix was constructed to describe the path relationships, with 0 indicating no correlation and 1 indicating correlation to characterize the correlation between latent variables. The strength of the correlation between the latent variables was quantified using the path coefficient. The fitting effect of the model was evaluated using goodness-of-fit measures. The closer the value is to 1, the better the fit. During model fitting, we gradually eliminate paths with low path coefficients or those that lead to overfitting to optimize the model structure.

## 3 Results

### 3.1 Changes in soil physical and chemical properties

In this study, a two-year (2023–2024) field fertilization experiment was conducted to assess the effects of different combined applications of nitrogen, phosphorus, and potassium on soil physical and chemical properties. The results showed that fertilization treatments showed significant interannual changes in multiple soil indicators. The soil bulk density in 2024 was generally higher than that in 2023, whereas the soil water content increased significantly in 2024. In terms of nutrient dynamics, available potassium showed a general downward trend over the years. In contrast, soil total phosphorus accumulated significantly. Except for the High- phosphorus (High-P) treatment, total phosphorus in the other treatments increased significantly in 2024 compared with 2023. It is worth noting that the soil calcium content decreased significantly from 2023 to 2024 across all treatments. Soil pH showed a significant upward trend in most treatments. Soil organic carbon fractions also varied between years: Organic carbon and mineral-associated organic carbon declined in some treatments, whereas organic matter increased significantly in 2024 compared with 2023. In most treatments, microbial biomass carbon and nitrogen increased significantly in 2024. At the same time, dissolved organic nitrogen and phosphorus also showed an upward trend. For the two nitrogen forms (NH_4_^+^-N and NO_3_-N), both increased significantly in both years, indicating that fertilization increased the content of available nitrogen in the soil (**Supplementary Table 2**).

To further distinguish the effects of fertilization treatment, year, and their interaction on soil physicochemical properties, a two-way ANOVA was conducted. The results showed that fertilization treatment had a significant effect on several soil properties, including bulk density, soil water content, available phosphorus, total phosphorus, pH, total dissolved nitrogen, dissolved organic nitrogen, dissolved organic phosphorus, particulate organic carbon, and microbial biomass carbon, indicating that fertilization effectively altered soil physicochemical conditions. In contrast, available potassium, total nitrogen, total potassium, calcium, and several organic carbon and nitrogen fractions (e.g., SOC, MAOC, MBN, NH_4_^+^-N, NO_3_^-^-N, and SOM) were not significantly affected by treatment, suggesting that these indicators were more strongly governed by interannual variation than by fertilization. Notably, bulk density, soil water content, available phosphorus, total phosphorus, pH, total dissolved nitrogen, dissolved organic nitrogen, dissolved organic phosphorus, and microbial biomass carbon were significantly affected by the year × treatment interaction, indicating that the response of these properties to fertilization differed between the two years. This interaction effect explains the significant interannual differences in soil nutrient levels described above and reflects the combined influence of the cumulative effects of fertilization and associated biological processes (**Supplementary Table 3**).

### 3.2 Changes in agronomic traits of alfalfa

**Figure 2:**
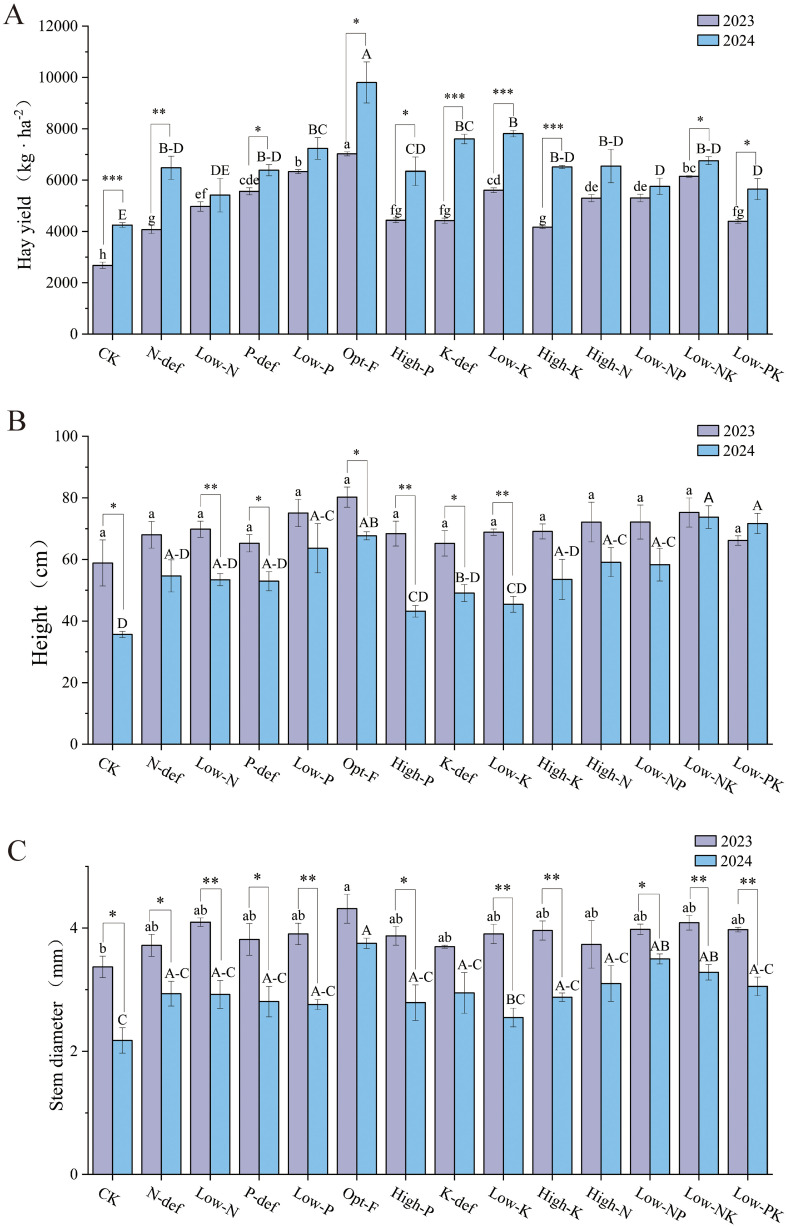
Effects of fertilization treatments in different years on agronomic traits of alfalfa. **(A)** Hay yield; **(B)** height; **(C)** stem diameter. CK, control; N-def, nitrogen-deficient treatment; Low-N, low-nitrogen treatment; P-def, phosphorus-deficient treatment; Low-P, low-phosphorus treatment; Opt-F, optimal fertilization treatment; High-P, high-phosphorus treatment; K-def, potassium-deficient treatment; Low-K, low-potassium treatment; High-K, high-potassium treatment; High-N, high-nitrogen treatment; Low-NP, low-nitrogen and low-phosphorus treatment; Low-NK, low-nitrogen and low-potassium treatment; Low-PK, low-phosphorus and low-potassium treatment. Different letters indicate statistically significant differences among treatments at p < 0.05. Asterisks above the bars indicate significant differences between years within the same fertilization treatment. *, **, and *** indicate p < 0.05, p < 0.01, and p < 0.001, respectively.

Fertilization significantly increased alfalfa yield compared with the CK treatment. The yield-increasing effect of the Optimal fertilization (Opt-F) treatment was particularly prominent, significantly exceeding that of the other treatments. Except for Low-nitrogen (Low-N), Low-phosphorus (Low-P), High-nitrogen (High-N), and Low-nitrogen and -phosphorus (Low-NP), the yields of the other treatments in 2024 were significantly higher than those in 2023 (**Figure 2A**). There was no significant difference among fertilization treatments in plant height in 2023. By 2024, there were significant differences in plant height among treatments, with Opt-F showing the highest. In addition, in most treatments, the plant height in 2023 was significantly higher than in 2024 (**Figure 2B**). In terms of stem diameter, plants in the Opt-F treatment in 2023 had a significantly larger stem diameter than those in the CK treatment, and there was no significant difference between the other treatments and the CK treatment. In 2024, the stem diameter of plants in the Opt-F treatment remained the highest among all treatments and was significantly higher than that of the CK treatment. In summary, the Opt-F treatment showed the greatest improvement in alfalfa agronomic traits (**Figure 2C**).

A two-way ANOVA was performed to evaluate the effects of fertilization treatment, year, and their interaction on alfalfa agronomic traits. Fertilization treatment had a significant effect on all three agronomic traits—hay yield, plant height, and stem diameter—indicating that different fertilization treatments substantially influenced alfalfa growth and productivity. Year also had a significant effect on all three traits, reflecting interannual variation in growth performance. For the year × treatment interaction, a significant effect was detected for hay yield, indicating that the response of yield to fertilization differed between the two years, whereas no significant interaction was found for plant height or stem diameter, suggesting that the treatment effects on these two traits were relatively consistent across years (**Supplementary Table 4**).

### 3.3 Changes in the rhizosphere bacteria community in alfalfa

**Figure 3:**
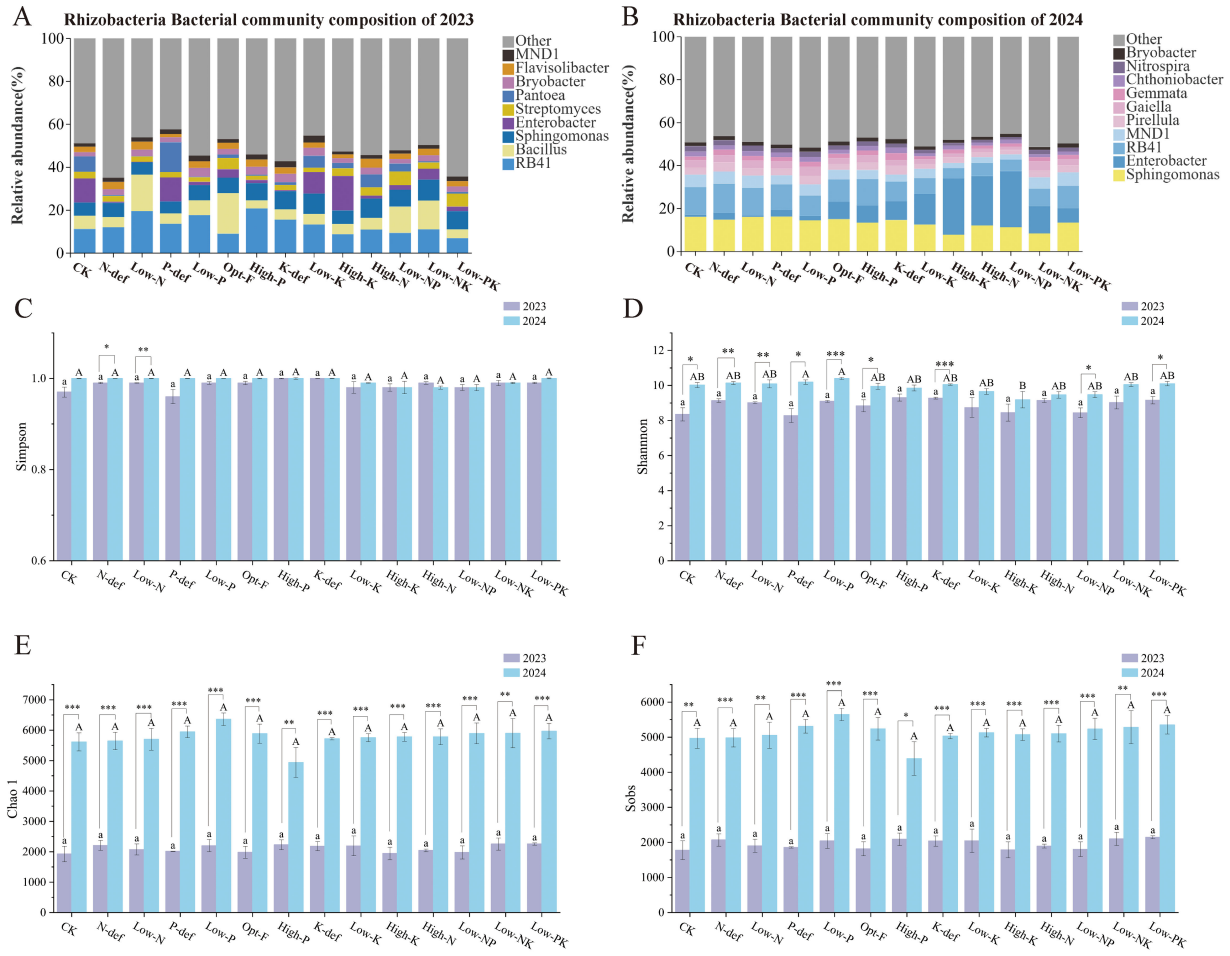
Effects of fertilization treatments in different years on the diversity of rhizosphere bacterial communities in alfalfa. **(A)** Rhizosphere bacterial community composition in 2023; **(B)** rhizosphere bacterial community composition in 2024; **(C)** Simpson index; **(D)** Shannon index; **(E)** Chao1 index; **(F)** Sobs index. CK, control; N-def, nitrogen-deficient treatment; Low-N, low-nitrogen treatment; P-def, phosphorus-deficient treatment; Low-P, low-phosphorus treatment; Opt-F, optimal fertilization treatment; High-P, high-phosphorus treatment; K-def, potassium-deficient treatment; Low-K, low-potassium treatment; High-K, high-potassium treatment; High-N, high-nitrogen treatment; Low-NP, low-nitrogen and low-phosphorus treatment; Low-NK, low-nitrogen and low-potassium treatment; Low-PK, low-phosphorus and low-potassium treatment. Different letters indicate statistically significant differences among treatments at p < 0.05. Asterisks above the bars indicate significant differences between years within the same fertilization treatment. *, **, and *** indicate p < 0.05, p < 0.01, and p < 0.001, respectively.

After excluding unclassified bacteria, RB41, *Bacillus*, *Sphingomonas*, and *Enterobacter* were the dominant genera in the alfalfa rhizosphere bacterial community in 2023. By 2024, the community composition had changed, with the dominant genera becoming *Sphingomonas*, *Enterobacter*, RB41, and MND1. Although the composition of the dominant bacteria across different fertilization treatments was the same, their relative abundances differed (**Figures 3A, B**).

In this study, the effects of fertilization on the four diversity indices of the rhizosphere bacterial community were analyzed. The results showed that fertilization treatment had no significant effect on the Simpson index over two years (**Figure 3C**). In the interannual comparison, only the Nitrogen-deficient (N-def) and Low-N treatments showed significant differences, whereas the remaining treatments showed no significant changes between years. In 2023, fertilization had no significant effect on the Shannon, Chao1, or Sobs indices. In 2024, fertilization still had no significant effect on the Chao1 and Sobs indices but did have a significant effect on the Shannon index. In addition, the Chao1 and Sobs indices showed significant differences between years (**Figures 3D–F**).

A two-way ANOVA was further conducted to evaluate the effects of fertilization treatment, year, and their interaction on rhizosphere bacterial diversity (**Supplementary Table 5**). Year had a significant effect on all four diversity indices (Sobs, Shannon, Simpson, and Chao), indicating pronounced interannual variation in rhizosphere bacterial diversity. Fertilization treatment had a significant effect on the Shannon and Simpson indices, which incorporate community evenness, whereas the richness-based indices (Sobs and Chao) were not significantly affected by treatment. The year × treatment interaction was not significant for any of the four indices, suggesting that the effects of fertilization on bacterial diversity were generally consistent between the two years, while the magnitude of the diversity indices differed markedly between years (**Supplementary Table 5**).

The results of the 2023 principal component analysis showed a high degree of overlap in rhizosphere bacterial composition across treatments, indicating significant compositional similarity. However, in 2024, the bacterial composition between different treatments showed significant separation, especially in High-potassium (High-K), High-N, and Low-nitrogen and -phosphorus (Low-NP). This result indicated that fertilization significantly affected the composition of the rhizosphere bacterial community (**Supplementary Figures 1A, B**).

### 3.4 Rhizosphere bacteria co-occurrence network and topology structure changes

**Figure 4:**
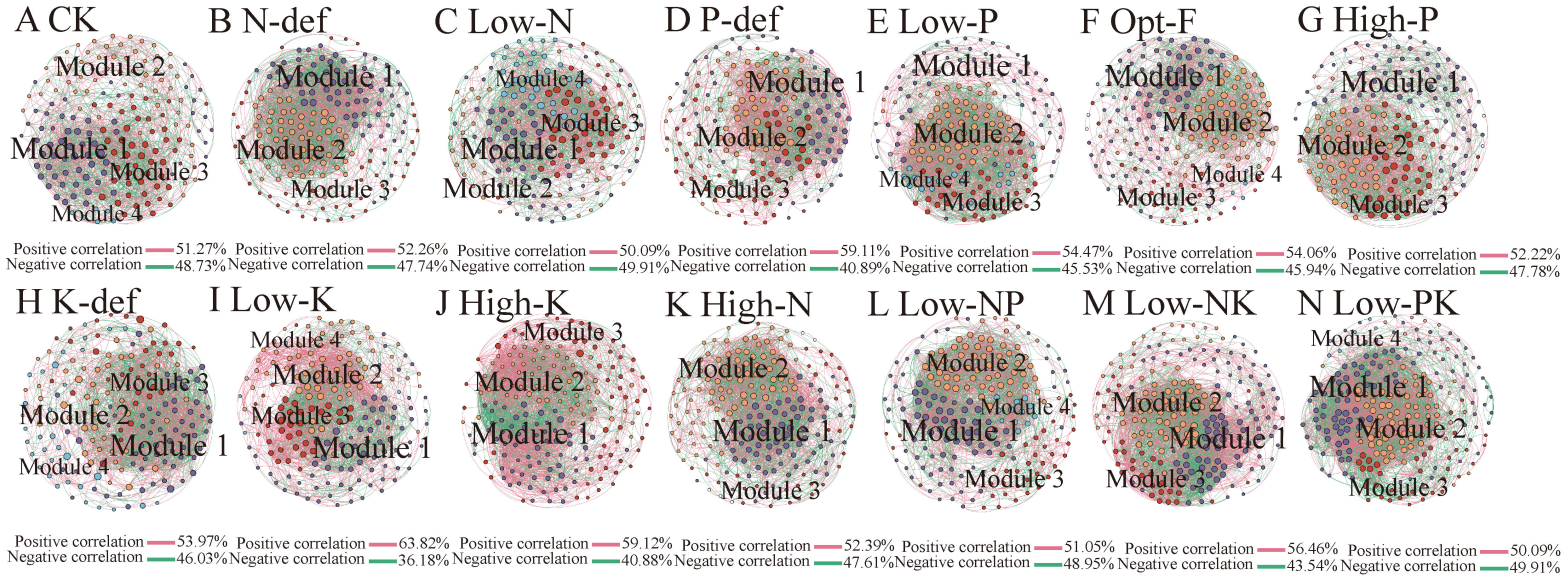
Effects of different fertilization measures on the co-occurrence network of rhizosphere bacteria in alfalfa. **(A)** CK; **(B)** N-def; **(C)** Low-N; **(D)** P-def; **(E)** Low-P; **(F)** Opt-F; **(G)** High-P; **(H)** K-def; **(I)** Low-K; **(J)** High-K; **(K)** High-N; **(L)** Low-NP; **(M)** Low-NK; **(N)** Low-PK. Nodes represent genus-level bacterial taxa, and edges represent significant Spearman correlations (|r| > 0.8, adjusted p < 0.05) between genera. Red and green edges denote positive and negative correlations, respectively. The percentages below each network indicate the proportion of positive and negative edges out of the total number of significant edges in that treatment’s subnetwork. CK, control; N-def, nitrogen-deficient treatment; Low-N, low-nitrogen treatment; P-def, phosphorus-deficient treatment; Low-P, low-phosphorus treatment; Opt-F, optimal fertilization treatment; High-P, high-phosphorus treatment; K-def, potassium-deficient treatment; Low-K, low-potassium treatment; High-K, high-potassium treatment; High-N, high-nitrogen treatment; Low-NP, low-nitrogen and low-phosphorus treatment; Low-NK, low-nitrogen and low-potassium treatment; Low-PK, low-phosphorus and low-potassium treatment.

Each treatment group showed a high degree of modularity in the analysis of the alfalfa rhizosphere bacteria co-occurrence network. The number of modules of N-def, Phosphorus-deficient (P-def), High-P, High-K, High-N, and Low-nitrogen and -potassium (Low-NK) decreased compared with CK. Additionally, fertilization affected the proportion of positive and negative interactions in the network. The other fertilization treatments increased the proportion of positive correlations to varying degrees, except for Low-N, Low-NP, and Low-phosphorus and -potassium (Low-PK), while the positive correlation ratio for the Low-potassium (Low-K) treatment was the highest among all treatments (**Figures 4A–N**).

**Figure 5:**
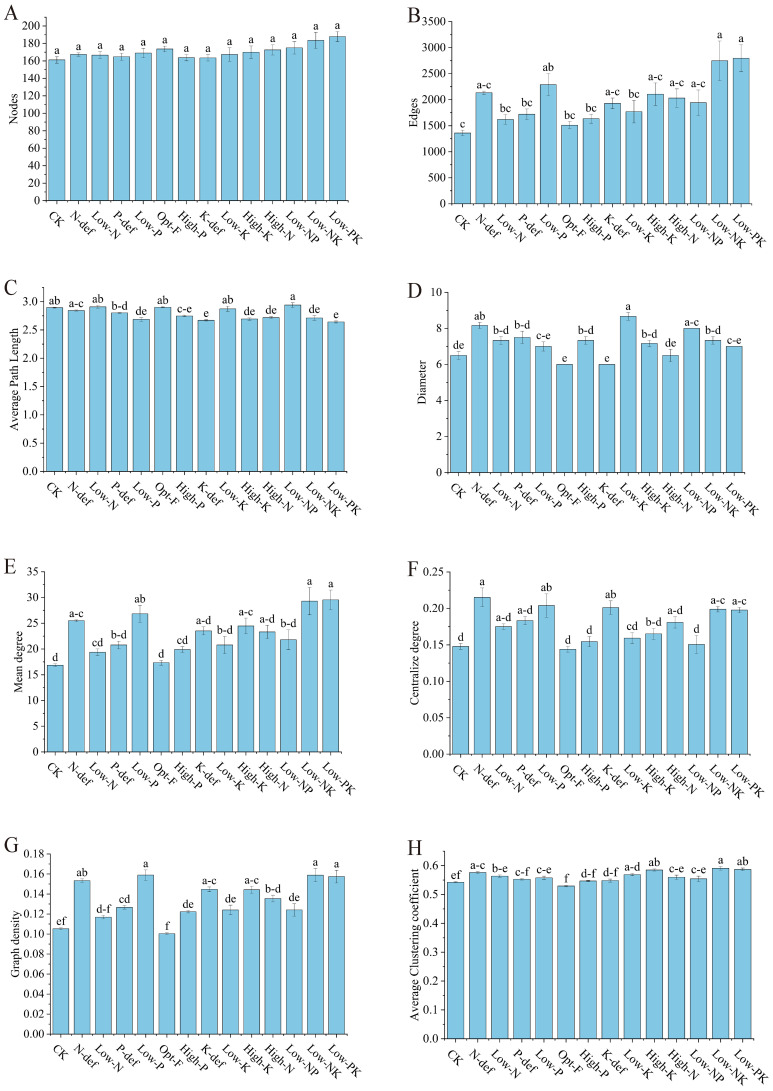
Effects of different fertilization measures on the co-occurrence network and topological parameters of alfalfa rhizosphere bacteria. **(A)** Nodes; **(B)** edges; **(C)** average path length; **(D)** diameter; **(E)** mean degree; **(F)** centralize degree; **(G)** graph density; **(H)** average clustering coefficient. CK, control; N-def, nitrogen-deficient treatment; Low-N, low-nitrogen treatment; P-def, phosphorus-deficient treatment; Low-P, low-phosphorus treatment; Opt-F, optimal fertilization treatment; High-P, high-phosphorus treatment; K-def, potassium-deficient treatment; Low-K, low-potassium treatment; High-K, high-potassium treatment; High-N, high-nitrogen treatment; Low-NP, low-nitrogen and low-phosphorus treatment; Low-NK, low-nitrogen and low-potassium treatment; Low-PK, low-phosphorus and low-potassium treatment.

This study further analyzed the effects of different fertilization treatments on the topological parameters of the bacteria co-occurrence network. The results showed that the fertilization treatment had no significant effect on the number of nodes but did affect other topological parameters. Specifically, fertilization significantly increased the number of network edges to varying degrees, especially in the Low-NK and Low-PK treatments. The average path length generally decreased significantly after fertilization. The network diameter did not show a consistent upward or downward trend; compared with CK, the diameter of the other treatments increased significantly, except for Opt-F and Potassium-deficient (K-def). In terms of mean degree, except for Opt-F, the other fertilization treatments increased significantly. The degree of centrality did not change significantly in the Opt-F, High-P, and Low-NP treatments, but increased significantly in the other treatments. Except for Opt-F, the other treatments significantly increased the graph density. Fertilization also increased the average clustering coefficient, whereas the Opt-F treatment showed a significantly reduced clustering coefficient (**Figures 5A–H**).

### 3.5 Correlation analysis of agronomic traits with bacteria and soil

**Figure 6:**
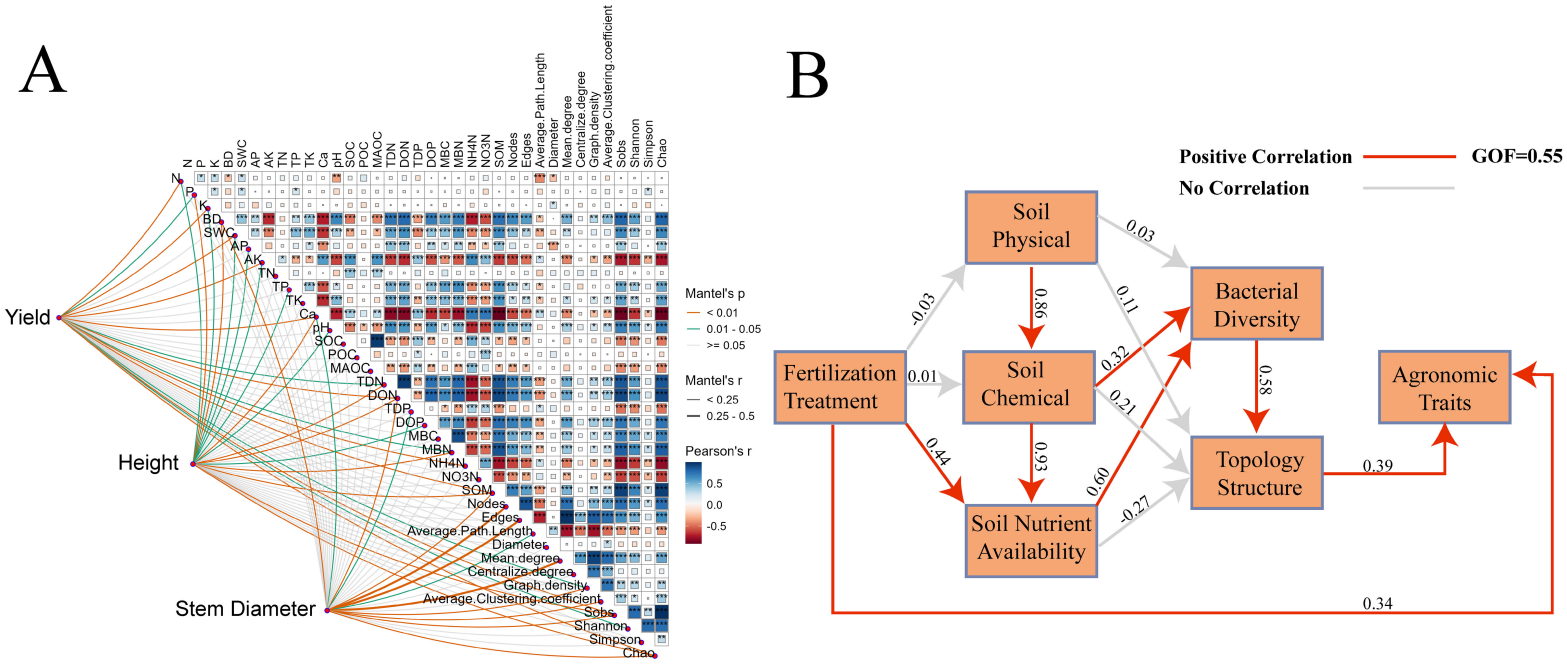
Correlation analysis between agronomic traits and factors of alfalfa. **(A)** Mantel’s test; **(B)** Partial least squares path analysis. N, Nitrogen fertilizer usage; P, Phosphate fertilizer usage; K, Potassium fertilizer usage; BD, Bulk density; SWC, soil water content; TN, soil total nitrogen; TP, soil total phosphorus; TK, soil total potassium; AP, available phosphorus; AK, available potassium; Ca, soil calcium; SOC, soil organic carbon; POC, particulate organic carbon; MAOC, mineral-associated organic carbon; TDN, total dissolved nitrogen; DON, dissolved organic nitrogen; TDP, total dissolved phosphorus; DOP, dissolved organic phosphorus; NH4+–N, Ammonium nitrogen; NO3—N, Nitrate nitrogen; SOM, soil organic matter; MBC, Microbial biomass carbo; MBN, Microbial biomass nitrogen; Agronomic traits: height, stem diameter, and yield.

The potential factors affecting alfalfa agronomic traits (height, stem diameter, and yield) were analyzed in this study using Mantel’s test. The results showed that the three agronomic traits were significantly correlated with fertilization treatment, soil physical and chemical properties, bacterial co-occurrence network topology parameters, and bacterial community diversity, indicating that improvements in alfalfa agronomic traits were affected by multiple factors (**Figure 6A**). A PLS-PM was used to fit the data and further explore the internal mechanisms by which fertilization treatment promotes alfalfa agronomic traits.

Fertilization significantly and directly improved alfalfa agronomic traits (path coefficient = 0.34). In addition, fertilization indirectly improved agronomic traits through a sequential mediating pathway: fertilization first enhanced soil nutrient availability (0.44), which significantly increased rhizosphere bacterial diversity (0.60); the increase in bacterial diversity, in turn, improved the topology of the bacterial co-occurrence network (0.58), and the optimized network topology ultimately promoted alfalfa agronomic traits (0.39). These results indicate that fertilization improved alfalfa agronomic traits through direct and indirect pathways, mediated by the soil nutrient–bacterial diversity network topology (**Figure 6B**).

## 4 Discussion

### 4.1 Potential mechanisms of soil physical and chemical changes and interannual differences

The results of this study showed that fertilization treatment significantly increased the content of available nutrients in the soil, mainly due to the direct input of fertilizer, which provided immediately available nutrients to the soil, as also demonstrated using PLS-SEM in this study (Yahaya et al., 2023; Jiang et al., 2024). The significant increase in soil organic carbon (SOC) and organic matter (SOM) may be related to the increase in plant root exudates, root residues, and aboveground litter input (Wu et al., 2022; Tang et al., 2025; Yao et al., 2026). Studies have confirmed that SOM can promote the formation of soil macroaggregates and increase porosity, thereby improving soil structure and enhancing water retention capacity, consistent with the increasing trend in soil water content (SWC) observed in this study (Lal, 2020). Additionally, appropriate fertilization can stimulate soil microbial activity and improve nutrient transformation efficiency. However, such biochemical processes often have a certain lag (Cookson et al., 2002). In this study, total dissolved nitrogen (TDN) and dissolved organic nitrogen (DON) did not differ significantly from CK in 2023. These variables were even lower than CK, whereas TDN and DON in each treatment were significantly higher than CK in 2024. At the same time, NH_4_^+^-N and NO_3_^--^N decreased in 2024 compared with 2023. This result indicates that nutrient inputs and early transformations may dominate the initial stage of the experiment (2023). In the next year (2024), the microbial-driven nitrogen transformation process is more efficient; hence, more nitrogen exists in soluble organic form, which promotes significant increases in active nitrogen components, such as DON and TDN. Microbial biomass carbon (MBC) and microbial biomass nitrogen (MBN) were significantly higher in 2024 than in 2023, further confirming that soil microbial biomass gradually increased with an increase in fertilization years, which was conducive to nutrient transformation and retention (Jones et al., 2004b; Schimel and Bennett, 2004; Spohn, 2015). Many other indicators also showed higher values in 2024 than in 2023. This result indicates that some nutrients in the fertilizers applied in the first year may be converted into stable organic forms or temporarily fixed by the soil. In the second year, these residual nutrients exerted a synergistic effect with the new fertilizer, jointly promoting further improvements in soil nutrient status (Yan et al., 2014).

### 4.2 The effect and mechanism of nitrogen, phosphorus, and potassium optimized ratio on significantly increasing alfalfa yield

During crop growth, nutrient availability in the environment directly affects growth and development (Nkebiwe et al., 2016). The application of fertilizers can rapidly increase the availability of soil nutrients and alleviate nutrient limitations on crop growth, thereby promoting leaf growth, enhancing photosynthetic capacity, stimulating root development, improving material transport efficiency, and ultimately increasing crop biomass and yield (Vejan et al., 2021). In this study, Opt-F (N2P2K2) treatment had the greatest effect on alfalfa yield, plant height, and stem diameter. This result is consistent with the explanation of “Liebig’s Law of the Minimum,” that is, nutrients with the lowest relative content limit crop yield (van der Ploeg et al., 1999; Agren et al., 2012). In soils with low basic fertility, the lack of nutrients can limit growth. The optimal fertilization treatment Opt-F simultaneously alleviated the potential limitations of the three nutrients by providing a balanced supply of nitrogen, phosphorus, and potassium and enabling their interaction. In summary, nitrogen promoted growth, phosphorus supplied energy, and assisted in transport, and potassium maintained physiological flow and health, thereby achieving an overall synergistic improvement in alfalfa growth traits (Venterink, 2016; Ringeval et al., 2021).

In addition to Opt-F, other treatments improved alfalfa agronomic traits to varying degrees; however, the increases were lower than those observed with Opt-F. This is because the fertilizer ratios in these treatments were suboptimal, and nutrient limitations or imbalances persisted after fertilization. For example, in Low-N (N0P2K2), although phosphorus and potassium contents increased, nitrogen deficiency made nitrogen the main limiting factor. Similarly, in treatments P-def and K-def, phosphorus and potassium deficiencies were the main limiting factors for alfalfa growth, respectively, resulting in lower alfalfa yields than in Opt-F (Gu et al., 2026). Additionally, in the “double low” treatment, both nutrients were simultaneously insufficient, and the limiting effect was more pronounced (Sun et al., 2025). Excessive application of a specific nutrient caused a nutrient imbalance in treatments High-P, High-K, and High-N, changing the fertilizer effect from promotion to inhibition and thereby affecting final yield (Albornoz, 2016; Song et al., 2022).

### 4.3 Interannual reconstruction of the rhizosphere bacteria community and network simplification under nutrient balance

The 2023 fertilization treatment did not significantly alter the diversity of rhizosphere bacterial communities, and there was no significant difference compared with the CK treatment.

The two-way ANOVA further showed that, although year had a significant effect on all four diversity indices, the year × treatment interaction was not significant, indicating that the diversity indices changed markedly between years in a manner that was generally consistent across treatments rather than being driven by treatment-specific responses to fertilization (**Supplementary Table 5**). The above interannual changes are mainly due to the following reasons. (1) The response of bacterial communities to environmental changes is often gradual and develops over time (Shade et al., 2012; Geisseler and Scow, 2014). Bacteria communities usually take a long time to reconstruct their niches after external disturbances. Additionally, soil and plants can buffer the effects of fertilization on bacteria. In the first year, fertilization was preferentially absorbed by plants or adsorbed by soil colloids, which may not immediately cause strong selection pressure on bacteria. As the experiment progressed into the second year, the cumulative effects of continued cultivation and fertilization gradually became apparent at the community level, accompanied by changes in bacterial community structure and diversity (Allison and Martiny, 2008; Griffiths and Philippot, 2013). (2) The enhancement of plant–microorganism interaction further strengthened the remodeling of the bacteria community. The root growth of alfalfa was significantly different between the first and second years of cultivation. The longer the plant grows, the more rhizosphere sediments (including root exudates, exfoliated cells, etc.) it transports into the soil. This new and stable carbon source input provides exclusive “food” for bacteria with specific functions, thus enabling directional enrichment of related bacterial groups and gradually building a new bacterial community over time. This phenomenon is more obvious in the rhizosphere (Badri et al., 2009; Philippot et al., 2013). (3) In previous studies, it was also found that the “historical effect” usually significantly affected the microbial community changes. Even though the diversity indices did not differ significantly among treatments in the first year, the early stage of cultivation and fertilization may have selectively stimulated or inhibited certain “keystone species,” gradually shifting the community toward a “new balance” that became detectable at the diversity level in the second year (Fukami, 2015; Banerjee et al., 2018).

By constructing the co-occurrence network of bacterial communities under different fertilization treatments and calculating the topological parameters, a seemingly contradictory phenomenon was observed in this study: except for the best fertilization treatment (Opt-F), the other fertilization treatments significantly increased the topological parameters of the network, indicating that the bacteria interaction network tended to be complicated. In contrast, the topological parameters of the Opt-F treatment, including edges, diameter, average degree, centralization, graph density, and average clustering coefficient, were significantly lower than those of the other treatments to varying degrees, indicating a clear trend toward simplification. The above phenomena may reflect a trade-off between the stability and complexity of bacterial network structures in response to environmental changes (Coyte et al., 2015). Under optimal fertilization treatment, nutrient supply is balanced and sufficient, allowing various bacterial groups to directly utilize abundant resources, thereby reducing dependence on interspecific interactions and the need to establish redundant connections. Also, the dominant flora may play a more efficient functional role under appropriate conditions without having to contend with environmental pressures through numerous complex collaborative relationships, thereby forming a simple yet efficient co-occurrence network (Wagg et al., 2014). Such simplified networks usually exhibit increased stability, reducing the risk of cascading failures following the failure of a single node and facilitating the efficient transmission of information and materials, thereby improving the response speed of the community to external changes (Paine et al., 2012). In contrast, in other fertilization treatments (such as nutrient deficiency or excess), the soil environment is disturbed to some extent, and bacteria are exposed to stressors, such as nutrient limitations or imbalances. Bacteria tend to strengthen their associations with one another, respond to adversity through mechanisms such as cross-feeding and co-metabolism, and may form many inefficient or redundant interactions as a buffer. These processes occur to maintain survival and function. These adaptive strategies ultimately result in a general increase in the topology parameters of the co-occurrence network and the overall complexity of the network structure (de Vries et al., 2018; Banerjee et al., 2019).

### 4.4 The synergistic mechanism of “plant–soil–microorganism” under fertilization treatment

In this study, the PLS-PM model was used to reveal the synergistic mechanism of plant–soil–microorganisms in the effects of fertilization on alfalfa agronomic traits. On the one hand, fertilization directly improved alfalfa agronomic traits; on the other hand, it indirectly increased bacterial diversity by increasing soil-available nutrient levels. Direct and indirect effects of soil physical and chemical properties regulate the availability of soil nutrients. The increase in bacterial diversity provides a basis for interspecific interactions, promotes collaboration and co-metabolism among bacteria, and thus significantly enhances the topological complexity of the rhizosphere bacteria co-occurrence network (Xu et al., 2013). Optimizing the network structure further improved the efficiency of soil nutrient acquisition and utilization by alfalfa, ultimately synergistically enhancing overall agronomic performance (Wagg et al., 2014; Shi et al., 2016).

## 5 Conclusion

Based on the “3414” field fertilization experiment conducted over two consecutive years, we examined the effects of different nitrogen, phosphorus, and potassium ratio fertilization on soil physical and chemical properties, agronomic traits of alfalfa, and the rhizosphere bacterial community. It incorporated the rhizosphere bacterial co-occurrence network into the “plant–soil–microorganism” analysis framework to reveal the internal relationship between the three. The results showed that the agronomic traits of alfalfa under the balanced fertilization treatment were significantly better than those under other treatments, and the rhizosphere bacterial network exhibited topological characteristics of “simple and efficient.” In contrast, nutrient limitation or imbalance treatment tended to be “complex redundancy.” The structural equation model further showed that fertilization promoted rhizosphere bacterial diversity by improving soil nutrient availability, thereby optimizing the co-occurrence network topology and ultimately enhancing alfalfa agronomic traits. This result indicates that a reasonable nutrient allocation ratio can directly increase yield and indirectly guarantee productivity by shaping a more stable and efficient rhizosphere microbial network. Therefore, in the large-scale production of alfalfa, a balanced ratio of nitrogen, phosphorus, and potassium should be given priority to avoid nutrient deficiency or imbalance. This study confirmed that the topological structure of the rhizosphere bacterial co-occurrence network is the key link between fertilization management and alfalfa agronomic traits, and provides a new perspective for field management and precision fertilization of alfalfa.

## Data Availability

The datasets presented in this study can be found in online repositories. The names of the repository/repositories and accession number(s) can be found below: https://www.ncbi.nlm.nih.gov/, PRJNA1438644.
