# Supplementary material for: Fertilization measures improve agronomic traits of alfalfa by regulating root bacterial co-occurrence networks
Source: Front Plant Sci. 2026 Jul 29;17:1882294. doi: 10.3389/fpls.2026.1882294 (PMC13461736; doi:10.3389/fpls.2026.1882294)
Supplement: Supplementary file 1 [file DataSheet1.docx]

Supplementary Material

# 1 Supplementary Figures and Tables

## 1.1 Supplementary Figures

**Figure.S1** PCA analysis of rhizosphere microorganisms in different years of fertilization. CK, control; N-def, nitrogen-deficient treatment; Low-N, low-nitrogen treatment; P-def, phosphorus-deficient treatment; Low-P, low-phosphorus treatment; Opt-F, optimal fertilization treatment; High-P, high-phosphorus treatment; K-def, potassium-deficient treatment; Low-K, low-potassium treatment; High-K, high-potassium treatment; High-N, high-nitrogen treatment; Low-NP, low-nitrogen and low-phosphorus treatment; Low-NK, low-nitrogen and low-potassium treatment; Low-PK, low-phosphorus and low-potassium treatment.

## 1.2 Supplementary Tables

**Table S1** Measurement methods of soil physical and chemical properties and biomass

| Soil indicators | Analytical methods | References |
| --- | --- | --- |
| Bulk density（BD） | cutting ring method | Xiao et al.，2020 |
| Soil water content (SWC) | Oven drying method | Cheng et al.，2025 |
| soil pH（pH） | pH meter | Le et al.，2025 |
| Soil total nitrogen (TN) | Kjeldahl method | Le et al.，2025 |
| total phosphorus (TP) | Akali fusion-Mo-Sb Anti method | Le et al.，2025 |
| soil total potassiumsoil(TK) | Flame atomic absorption spectrophotometer | Le et al.，2025 |
| available phosphorus (AP) | Molybdenum antimony colorimetry | Le et al.，2025 |
| available potassium (AK) | NH_4_OAc extraction and fame photometry | Le et al.，2025 |
| soil calcium(Ca) | Flame atomic absorption spectrophotometer | Le et al.，2025 |
| soil organic carbon (SOC) | Wet combustion procedure | Le et al.，2025 |
| particulate organic carbon (POC), | Sodium hexametaphosphate dispersion-external heating method | Cambardella et al.，1992 |
| mineral-associated organic carbon (MAOC) | Sodium hexametaphosphate dispersion-external heating method | Cambardella et al.，1992 |
| total dissolved nitrogen (TDN) | Distilled Water Extraction - TOC Analyzer Test Method | Gorbov S Net al., 2021 |
| dissolved organic nitrogen (DON) | Kjeldahl method | Le et al.，2025 |
| total dissolved phosphorus (TDP) | Molybdenum-Antimony Antimony Colorimetric Method | Kowalenko et al.，2007 |
| dissolved organic phosphorus (DOP) | total dissolved phosphorus (TDP) minus dissolved organic phosphorus (DOP) | Kowalenko et al.，2007 |
| Ammonium nitrogen (NH_4_^+^–N) | Potassium chloride solution extraction method | Kachurina et al.，2000 |
| Nitrate nitrogen (NO_3_^−^–N) | Potassium chloride solution extraction method | Kachurina et al.，2000 |
| soil organic matter (SOM) | Potassium dichromate oxidation external heating method | Wu et al.，2017 |
| Microbial biomass carbon (MBC) | Chloroform fumigation extraction method | Le et al.，2025 |
| Microbial biomass nitrogen (MBN) | Chloroform fumigation extraction method | Le et al.，2025 |

**References**

Xiao, L., Yuan, G., Feng, L., et al., 2020. Coupled effects of biochar use and farming practice on physical properties of a salt-affected soil with wheat–maize rotation. Journal of Soils and Sediments 20, 3053-3061.

Cheng, J., Zheng, K., Xia, J., et al., 2025. Effects of ultrasonic vibration-assisted cutting on tillage blade performance and soil fragmentation characteristics under different soil moisture contents. Soil and Tillage Research 252, 106575.

Le, Q., Ru, M., Weibo, R., et al., 2025. Biological crusts increase soil microbial biomass and alter bacterial and fungal community compositions under pine restored over ten years in a Pisha sandstone soil. Frontiers in Ecology and Evolution 13, 1524202.

Cambardella, C.A., Elliott, E.T., 1992. Particulate soil organic-matter changes across a grassland cultivation sequence. Soil Science Society of America Journal 56, 777-783.

Kachurina, O.M., Zhang, H., Raun, W.R., et al., 2000. Simultaneous determination of soil aluminum, ammonium- and nitrate-nitrogen using 1 M potassium chloride extraction. Communications in Soil Science and Plant Analysis 31, 893-903.

Wu, Q., Liang, Y., Li, Y., et al., 2017. Factors acquisition and content estimation of farmland soil organic carbon based upon Internet of Things. Chinese Geographical Science 27, 431-440.

Kowalenko, C.G., Babuin, D., 2007. Interference problems with phosphoantimonylmolybdenum colorimetric measurement of phosphorus in soil and plant materials. Communications in Soil Science and Plant Analysis 38, 1299-1316.

Gorbov, S.N., Gorovtsov, A.V., Bezuglova, O.S., et al., 2021. Enzyme activity of soils in urban landscapes of the lower Don area, Southern Russia. Land Degradation & Development 32, 1618-1631.

Yun, A., Shi, S., Gong, W., et al., 2022. Cross-breeding improvement and performance analysis of dominant production traits in grazing-type alfalfa (Medicago sativa L.). Biomed Research International 2022, 1252310.

Kusi, N.Y.O., Temu, V.W., Kering, M.K., et al., 2021. Growth response of alfalfa to Azomite composite micronutrient fertilizer on four lime-amended Virginia soils. Grassland Science 67, 225-233.

Zhang, T., Wang, X., Han, J., et al., 2008. Effects of between-row and within-row spacing on alfalfa seed yields. Crop Science 48, 794-803.

Peng, X., Sun, Q., Zhang, S., et al., 2025. A Comparative Analysis of the Efficacy of Three Plant Growth Regulators and Dose Optimization for Improving Agronomic Traits and Seed Yield of Purple-Flowered Alfalfa (Medicago sativa L.). Plants 14, 2258.

**Table S2** Effects of different fertilization treatments and years on soil physicochemical properties.

| Soil properties | Treatment | CK | N-def | Low-N | P-def | Low-P | Opt-F | High-P | K-def | Low-K | High-K | High-N | Low-NP | Low-NK | Low-PK |
| --- | --- | --- | --- | --- | --- | --- | --- | --- | --- | --- | --- | --- | --- | --- | --- |
| BD(g·cm^-3^) | 2023 | 1.33±0.04a | 1.25±0.01ab | 1.22±0.00ab | 1.24±0.04ab | 1.14±0.01b | 1.15±0.02b | 1.24±0.01ab | 1.19±0.02ab | 1.25±0.04ab | 1.19±0.06ab | 1.18±0.06ab | 1.29±0.03ab | 1.31±0.04ab | 1.16±0.03ab |
|  | 2024 | 1.49±0.01A-E* | 1.49±0.04A-E** | 1.37±0.02EF** | 1.43±0.03B-E* | 1.40±0.02C-E*** | 1.39±0.02D-F** | 1.27±0.02F | 1.50±0.01A-D*** | 1.44±0.02B-E* | 1.47±0.04A-E* | 1.43±0.02B-E* | 1.58±0.01A*** | 1.52±0.03A-C* | 1.54±0AB*** |
| SWC(%) | 2023 | 7.51±0.94b | 8.34±0.38ab | 9.27±0.66ab | 8.70±0.22ab | 8.96±0.09ab | 10.10±1.12ab | 9.05±1.21ab | 12.99±0.73a | 10.79±0.48ab | 11.54±2.24ab | 8.65±0.17ab | 8.32±0.53ab | 8.58±0.71ab | 7.99±0.97b |
|  | 2024 | 11.84±0.08H* | 12.46±0.11F-H*** | 13.51±0.34EF** | 9.91±0.17I* | 15.97±0.05B*** | 15.57±0.12BC** | 14.72±0.33CD* | 12.50±0.07F-H | 16.51±0.48B** | 17.73±0.07A | 13.25±0.02E-G*** | 12.31±0.38GH** | 8.70±0.18J | 14.03±0.07DE** |
| AP(mg·kg^-1^) | 2023 | 7.43±0.76a | 5.9±1.00a | 5.74±0.69a | 5.22±0.59a | 6.57±0.88a | 8.33±0.32a | 5.02±0.37a | 6.34±1.11a | 4.20±0.05a | 6.71±2.72a | 6.06±1.15a | 5.08±0.30a | 5.91±0.80a | 5.94±0.24a |
|  | 2024 | 12.37±3.55A-C | 5.58±0.27BC | 6.04±0.45BC | 10.46±3.43A-C | 4.96±0.38BC | 11.00±1.16A-C | 7.22±1.01BC | 20.25±5.16A | 10.60±0.91A-C** | 14.67±2.04AB | 8.85±0.19BC | 8.37±0.22BC*** | 7.83±0.83BC | 3.56±1.16C |
| AK(mg·kg^-1^) | 2023 | 250.12±35.50a | 260.53±19.09a | 254.31±6.88a | 251.89±20.55a | 227.05±23.13a | 266.68±32.32a | 240.08±34.00a | 245.26±47.52a | 187.44±12.85a | 303.45±19.64a | 255.00±20.79a | 257.45±27.24a | 198.51±37.61a | 230.51±25.10a |
|  | 2024 | 164.33±28.48A | 129.24±9.66A** | 159.87±17.42A** | 148.35±14.47A* | 186.82±11.79A | 164.76±6.60A* | 163.56±26.69A | 156.58±13.94A | 164.25±25.89A | 152.14±16.15A** | 161.61±15.85A* | 150.97±13.52A* | 145.85±21.51A | 145.35±11.86A* |
| TN(g·kg^-1^) | 2023 | 1.25±0.08a | 1.32±0.05a | 1.30±0.05a | 1.14±0.04a | 1.06±0.11a | 1.36±0.25a | 1.33±0.18a | 1.22±0.10a | 1.31±0.04a | 1.36±0.08a | 1.34±0.02a | 1.05±0.08a | 1.18±0.07a | 1.21±0.07a |
|  | 2024 | 1.14±0.14A | 1.01±0.33A | 0.93±0.32A | 1.48±0.26A | 1.48±0.08A* | 0.68±0.30A | 1.37±0.16A | 1.25±0.09A | 1.51±0.25A | 1.24±0.09A | 1.02±0.30A | 0.94±0.31A | 1.35±0.19A | 1.36±0.05A |
| TP(mg·kg^-1^) | 2023 | 741.00±11.75a | 692.36±16.01a | 745.45±42.70a | 731.69±6.05a | 750.84±4.20a | 729.93±7.8a | 730.54±14.05a | 727.85±17.38a | 743.91±24.04a | 766.10±45.19a | 737.39±28.11a | 751.39±29.25a | 739.17±12.94a | 732.00±25.99a |
|  | 2024 | 804.44±22.27B | 804.86±21.00B* | 862.38±35.95B | 821.20±23.88B* | 850.78±23.82B* | 841.36±12.85B** | 1215.02±185.36A | 833.88±9.92B** | 894.00±24.39B* | 814.34±9.60B | 847.34±10.61B* | 895.45±38.30B* | 852.47±14.52B** | 827.66±16.53B* |
| TK(g·kg^-1^) | 2023 | 16.85±0.55a | 17.04±0.35a | 19.10±0.77a | 17.92±0.99a | 20.31±0.65a | 17.27±1.47a | 18.25±0.77a | 19.06±1.67a | 19.17±2.61a | 18.93±0.94a | 16.85±1.24a | 17.01±0.64a | 16.46±1.27a | 18.03±1.14a |
|  | 2024 | 20.55±0.27A** | 20.34±0.07A*** | 20.73±0.18A | 20.13±0.19A | 20.41±0.11A | 20.39±0.08A | 20.11±0.31A | 20.17±0.06A | 20.23±0.28A | 20.26±0.12A | 20.41±0.33A | 20.33±0.22A** | 19.95±0.31A | 20.30±0.31A |
| Ca(g·kg^-1^) | 2023 | 44.77±3.35a | 42.26±0.76a | 43.13±0.60a | 47.65±3.19a | 44.34±0.63a | 45.02±2.28a | 47.18±0.75a | 46.70±1.96a | 45.04±0.68a | 48.02±0.46a | 49.29±2.67a | 54.76±7.49a | 50.51±3.90a | 50.57±3.98a |
|  | 2024 | 25.46±1.45A** | 25.81±0.31A*** | 25.41±1.10A*** | 29.32±2.05A** | 25.11±0.96A*** | 24.31±0.58A*** | 26.64±1.28A*** | 26.32±1.30A*** | 25.49±1.31A*** | 26.51±1.56A*** | 27.14±3.26A** | 28.08±2.40A* | 28.88±2.50A** | 27.50±2.83A** |
| pH | 2023 | 8.15±0.04ab | 8.18±0.02a | 8.11±0.02a-c | 8.07±0.03a-d | 8.11±0.03a-c | 8.01±0.04b-e | 8.05±0.02a-d | 7.97±0.05c-e | 8.00±0.06b-e | 7.93±0.05de | 7.92±0.02de | 7.85±0.00e | 7.99±0.03b-e | 7.93±0.01de |
|  | 2024 | 8.28±0.02A* | 8.29±0.03A* | 8.29±0.03A* | 8.23±0.04A* | 8.23±0.04A | 8.20±0.02A** | 8.11±0.09A | 8.13±0.01A* | 8.11±0.04A | 8.19±0.01A** | 8.17±0.03A** | 8.28±0.07A** | 8.26±0.04A** | 8.21±0.07A* |
| SOC(g·kg^-1^) | 2023 | 17.19±0.30a | 16.03±1.26a | 17.25±0.34a | 16.60±1.44a | 17.21±0.15a | 16.58±0.21a | 16.71±1.64a | 18.71±0.21a | 16.22±1.61a | 17.08±1.09a | 14.87±2.12a | 17.23±1.52a | 15.10±0.86a | 18.98±0.41a |
|  | 2024 | 13.43±0.83A* | 12.52±0.77A | 13.89±1.09A* | 15.00±3.02A | 15.96±1.05A | 13.33±1.46A | 14.74±1.95A | 14.39±1.65A | 16.19±2.72A | 13.13±1.15A | 14.19±1.69A | 13.89±0.90A | 14.42±2.05A | 14.00±1.28A* |
| POC(g·kg^-1^) | 2023 | 2.07±0.31ab | 1.52±0.35b | 2.37±0.27ab | 2.06±0.28ab | 2.26±0.45ab | 1.93±0.08ab | 1.84±0.33b | 2.67±0.21ab | 4.00±0.16a | 2.17±0.87ab | 2.90±0.08ab | 2.31±0.53ab | 2.83±0.41ab | 2.15±0.54ab |
|  | 2024 | 1.87±0.30AB | 1.63±0.13AB | 1.85±0.09AB | 3.34±0.88A | 2.09±0.16AB | 2.08±0.39AB | 2.21±0.19AB | 1.65±0.20AB* | 2.51±0.43AB* | 1.51±0.27B | 2.08±0.17AB* | 2.25±0.25AB | 2.76±0.43AB | 2.45±0.11AB |
| MAOC(g·kg^-1^) | 2023 | 15.12±0.27a | 14.51±1.60a | 14.88±0.61a | 14.54±1.40a | 14.95±0.42a | 14.64±0.22a | 14.87±1.44a | 16.03±0.41a | 12.22±1.67a | 14.91±1.81a | 11.96±2.15a | 14.91±2.03a | 12.28±1.10a | 16.84±0.29a |
|  | 2024 | 11.55±0.63A** | 10.89±0.90A | 12.04±1.14A | 11.65±2.15A | 13.86±1.01A | 11.26±1.73A | 12.53±1.87A | 12.73±1.85A | 13.68±2.65A | 11.63±1.39A | 12.11±1.82A | 11.64±0.89A | 11.66±2.36A | 11.55±1.25A* |
| TDN(mg·kg^-1^) | 2023 | 13.19±0.88b | 18.31±0.37a | 13.21±0.41b | 12.75±0.07b | 10.68±0.46b-d | 8.52±1.11c-f | 8.90±0.75c-e | 5.07±0.00fg | 7.23±0.43d-f | 6.82±0.83ef | 8.55±0.40c-f | 5.54±0.43ef | 1.69±0.42g | 11.05±1.69bc |
|  | 2024 | 23.85±0.46C*** | 21.92±0.45C** | 20.37±4.06C | 30.53±2.10A-C** | 24.44±0.87A-C*** | 23.27±2.64C** | 24.24±1.02BC*** | 30.16±2.22A-C*** | 35.92±2.67A*** | 30.58±3.22A-C** | 35.50±3.10AB*** | 27.00±2.43A-C*** | 31.13±0.59A-C*** | 24.46±1.57A-C** |
| DON(mg·kg^-1^) | 2023 | 0.95±0.19ab | 1.07±0.49ab | 1.01±0.40ab | 1.97±0.65a | 1.30±0.14ab | 1.55±0.24ab | 1.93±0.35a | 1.04±0.11ab | 0.91±0.14ab | 1.23±0.09ab | 1.18±0.26ab | 1.22±0.16ab | 0.19±0.04b | 0.88±0.17ab |
|  | 2024 | 16.68±0.17BC*** | 14.84±0.75C*** | 12.88±3.15C* | 18.46±2.51A-C** | 15.08±0.66BC*** | 13.76±2.04C** | 15.80±0.42BC*** | 21.46±2.22A-C*** | 25.74±1.99AB*** | 22.19±2.80A-C** | 27.83±3.25A** | 18.67±2.24A-C** | 22.43±1.2A-C*** | 16.56±2.51BC** |
| TDP(mg·kg^-1^) | 2023 | 1.14±0.01a | 1.23±0.12a | 1.24±0.30a | 1.07±0.07a | 1.06±0.06a | 1.16±0.01a | 1.01±0.01a | 1.04±0.03a | 1.09±0.03a | 1.02±0.02a | 0.99±0.05a | 1.23±0.02a | 1.13±0.08a | 1.19±0.01a |
|  | 2024 | 0.73±0.04A*** | 0.72±0.09A* | 0.96±0.18A | 0.86±0.19A | 0.86±0.08A | 0.75±0.07A** | 0.92±0.14A | 0.85±0.03A** | 1.04±0.09A | 0.72±0.06A** | 0.98±0.05A | 1.27±0.20A | 1.20±0.11A | 0.70±0.12A* |
| DOP(mg·kg^-1^) | 2023 | 0.12±0.01a | 0.13±0.00a | 0.12±0.01a | 0.12±0.01a | 0.10±0.00a | 0.11±0.01a | 0.10±0.01a | 0.11±0.01a | 0.10±0.01a | 0.12±0.01a | 0.12±0.01a | 0.13±0.01a | 0.11±0.01a | 0.12±0.01a |
|  | 2024 | 0.39±0.09AB* | 0.44±0.12AB | 0.68±0.20AB* | 0.54±0.22AB | 0.63±0.09AB** | 0.28±0.03B** | 0.50±0.20AB | 0.66±0.04AB*** | 0.64±0.09AB** | 0.50±0.08AB* | 0.79±0.08AB** | 1.09±0.25A* | 1.07±0.08A*** | 0.54±0.17AB |
| MBC(mg·kg^-1^) | 2023 | 160.65±17.71ab | 156.56±29.75ab | 150.56±11.61ab | 110.32±41.68b | 152.19±3.42ab | 112.35±34.94b | 203.85±36.39ab | 145.97±17.46b | 185.73±6.48ab | 200.07±24.55ab | 169.72±18.83ab | 237.60±42.04ab | 278.51±9.66a | 221.78±6.04ab |
|  | 2024 | 289.87±27.38A* | 269.64±8.35A* | 242.88±40.52A | 342.34±56.48A* | 351.56±30.18A** | 287.03±6.98A** | 308.87±53.53A | 259.36±15.26A** | 367.67±53.67A* | 249.49±17.45A | 245.02±8.98A* | 253.99±19.43A | 332.28±28.04A | 276.02±19.23A |
| MBN(mg·kg^-1^) | 2023 | 11.60±3.08ab | 19.13±9.64ab | 10.51±2.82ab | 5.40±0.61b | 7.98±3.72ab | 9.42±2.44ab | 17.91±4.17ab | 11.73±2.34ab | 19.16±0.72ab | 19.49±2.17ab | 17.38±1.85ab | 14.75±3.20ab | 23.92±0.28a | 11.26±2.60ab |
|  | 2024 | 49.58±6.33A** | 49.33±7.77A | 39.14±8.38A* | 60.95±12.85A* | 58.16±6.09A** | 52.12±4.13A*** | 58.70±14.56A | 48.02±4.04A** | 71.22±16.60A* | 45.53±4.85A** | 41.83±2.67A** | 47.14±6.52A* | 54.55±5.81A** | 41.35±1.19A*** |
| NH_4_^+^-N(mg·kg^-1^) | 2023 | 5.24±0.65a | 4.59±0.09a | 4.71±0.06a | 4.42±0.03a | 4.88±0.05a | 4.80±0.12a | 4.85±0.14a | 5.52±0.60a | 5.45±0.19a | 5.59±0.30a | 5.19±0.22a | 4.91±0.23a | 4.93±0.07a | 5.25±0.02a |
|  | 2024 | 3.87±0.08A | 3.84±0.04A** | 3.96±0.18A* | 3.86±0.09A** | 3.91±0.06A*** | 4.24±0.15A* | 3.83±0.16A** | 3.87±0.11A | 4.10±0.08A** | 3.81±0.11A** | 3.81±0.07A** | 3.86±0.06A* | 3.81±0.14A** | 3.79±0.16A*** |
| NO_3_^-^-N(mg·kg^-1^) | 2023 | 8.75±0.91a | 8.63±0.68a | 5.80±1.22a | 12.96±2.35a | 14.98±3.89a | 12.06±1.07a | 10.00±1.30a | 9.45±2.10a | 12.19±3.27a | 10.60±4.31a | 9.59±1.68a | 15.91±8.26a | 10.66±1.48a | 16.06±2.76a |
|  | 2024 | 3.31±0.29B** | 3.24±0.52B** | 3.54±0.77AB | 8.21±2.18A | 5.45±0.64AB | 5.27±0.62AB** | 4.61±0.90AB* | 4.84±0.53AB | 6.08±1.27AB | 4.58±0.66AB | 3.87±0.24AB* | 4.47±0.90AB | 4.89±0.81AB* | 4.11±0.81AB* |
| SOM（g·kg^-1^) | 2023 | 2.97±0.05a | 2.77±0.22a | 2.97±0.06a | 2.86±0.25a | 2.97±0.02a | 2.86±0.04a | 2.88±0.29a | 3.22±0.04a | 2.79±0.28a | 2.94±0.19a | 2.56±0.36a | 2.97±0.26a | 2.61±0.15a | 3.27±0.07a |
|  | 2024 | 23.15±1.43A*** | 21.58±1.33A*** | 23.94±1.88A*** | 25.85±5.21A* | 27.52±1.81A*** | 22.99±2.52A** | 25.42±3.36A** | 24.81±2.84A** | 27.91±4.69A** | 22.64±1.99A*** | 24.47±2.91A** | 23.95±1.55A*** | 24.86±3.54A** | 24.14±2.20A*** |

Mean bulk density (BD), soil water content (SWC), soil pH, soil total nitrogen (TN), total phosphorus (TP), soil total potassiumsoil (TK), available phosphorus (AP), available potassium (AK), soil calcium(Ca), soil organic carbon (SOC), particulate organic carbon (POC), mineral-associated organic carbon (MAOC), total dissolved nitrogen (TDN), dissolved organic nitrogen (DON), total dissolved phosphorus (TDP), dissolved organic phosphorus (DOP), Ammonium nitrogen (NH4+–N), Nitrate nitrogen (NO3−–N), soil organic matter (SOM), Microbial biomass carbon (MBC), Microbial biomass nitrogen (MBN), lowercase letters indicate statistically significant differences among treatments in the first year; uppercase letters indicate significant differences in the second year (p < 0.05). Asterisks denote significant differences between fertilization years. *, **, and *** represent p < 0.05, p < 0.01, and p < 0.001, respectively., and the data are presented as mean ± standard error.

**Table S3** Two-way ANOVA results for the effects of fertilization treatment, year, and their interaction on soil physicochemical properties.

| Index | df | Sum of Squares | Mean Square | F value | P value | Sig | Index | df | Sum of Squares | Mean Square | F value | P value | Sig |
| --- | --- | --- | --- | --- | --- | --- | --- | --- | --- | --- | --- | --- | --- |
| BD | 1 | 1.090 | 1.090 | 418.961 | 0.000 | *** | MAOC | 1 | 122.839 | 122.839 | 18.566 | 0.000 | *** |
|  | 13 | 0.245 | 0.019 | 7.251 | 0.000 | *** |  | 13 | 44.310 | 3.408 | 0.515 | 0.906 | ns |
|  | 13 | 0.135 | 0.010 | 3.997 | 0.000 | *** |  | 13 | 61.787 | 4.753 | 0.718 | 0.738 | ns |
|  | 56 | 0.146 | 0.003 |  |  |  |  | 56 | 370.520 | 6.616 |  |  |  |
| SWC | 1 | 363.210 | 363.210 | 270.923 | 0.000 | *** | TDN | 1 | 6795.363 | 6795.363 | 811.410 | 0.000 | *** |
|  | 13 | 229.663 | 17.666 | 13.178 | 0.000 | *** |  | 13 | 355.036 | 27.310 | 3.261 | 0.001 | ** |
|  | 13 | 103.697 | 7.977 | 5.950 | 0.000 | *** |  | 13 | 1284.148 | 98.781 | 11.795 | 0.000 | *** |
|  | 56 | 75.076 | 1.341 |  |  |  |  | 56 | 468.987 | 8.375 |  |  |  |
| AP | 1 | 239.811 | 239.811 | 29.853 | 0.000 | *** | DON | 1 | 6482.100 | 6482.100 | 963.157 | 0.000 | *** |
|  | 13 | 437.009 | 33.616 | 4.185 | 0.000 | *** |  | 13 | 385.194 | 29.630 | 4.403 | 0.000 | *** |
|  | 13 | 348.674 | 26.821 | 3.339 | 0.001 | *** |  | 13 | 432.736 | 33.287 | 4.946 | 0.000 | *** |
|  | 56 | 449.852 | 8.033 |  |  |  |  | 56 | 376.883 | 6.730 |  |  |  |
| AK | 1 | 163308.479 | 163308.479 | 99.405 | 0.000 | *** | TDP | 1 | 0.999 | 0.999 | 29.278 | 0.000 | *** |
|  | 13 | 16692.849 | 1284.065 | 0.782 | 0.676 | ns |  | 13 | 0.812 | 0.062 | 1.831 | 0.060 | ns |
|  | 13 | 21909.116 | 1685.317 | 1.026 | 0.441 | ns |  | 13 | 0.723 | 0.056 | 1.630 | 0.104 | ns |
|  | 56 | 92000.273 | 1642.862 |  |  |  |  | 56 | 1.911 | 0.034 |  |  |  |
| TN | 1 | 0.052 | 0.052 | 0.545 | 0.463 | ns | DOP | 1 | 5.483 | 5.483 | 181.447 | 0.000 | *** |
|  | 13 | 1.096 | 0.084 | 0.892 | 0.566 | ns |  | 13 | 1.067 | 0.082 | 2.715 | 0.005 | ** |
|  | 13 | 1.801 | 0.139 | 1.467 | 0.160 | ns |  | 13 | 1.035 | 0.080 | 2.636 | 0.006 | ** |
|  | 56 | 5.289 | 0.094 |  |  |  |  | 56 | 1.692 | 0.030 |  |  |  |
| TP | 1 | 364938.398 | 364938.398 | 69.966 | 0.000 | *** | MBC | 1 | 270915.559 | 270915.559 | 108.346 | 0.000 | *** |
|  | 13 | 214427.711 | 16494.439 | 3.162 | 0.001 | ** |  | 13 | 78085.741 | 6006.595 | 2.402 | 0.012 | * |
|  | 13 | 214683.650 | 16514.127 | 3.166 | 0.001 | ** |  | 13 | 79035.015 | 6079.617 | 2.431 | 0.011 | * |
|  | 56 | 292091.825 | 5215.925 |  |  |  |  | 56 | 140025.541 | 2500.456 |  |  |  |
| TK | 1 | 110.126 | 110.126 | 48.351 | 0.000 | *** | MBN | 1 | 28747.150 | 28747.150 | 227.123 | 0.000 | *** |
|  | 13 | 28.081 | 2.160 | 0.948 | 0.512 | ns |  | 13 | 2186.672 | 168.206 | 1.329 | 0.225 | ns |
|  | 13 | 25.611 | 1.970 | 0.865 | 0.593 | ns |  | 13 | 1944.443 | 149.573 | 1.182 | 0.317 | ns |
|  | 56 | 127.549 | 2.278 |  |  |  |  | 56 | 7087.958 | 126.571 |  |  |  |
| Ca | 1 | 8841.863 | 8841.863 | 473.629 | 0.000 | *** | NH_4_^+^-N | 1 | 26.747 | 26.747 | 202.269 | 0.000 | *** |
|  | 13 | 407.559 | 31.351 | 1.679 | 0.091 | ns |  | 13 | 2.598 | 0.200 | 1.511 | 0.142 | ns |
|  | 13 | 124.141 | 9.549 | 0.512 | 0.908 | ns |  | 13 | 2.923 | 0.225 | 1.700 | 0.086 | ns |
|  | 56 | 1045.427 | 18.668 |  |  |  |  | 56 | 7.405 | 0.132 |  |  |  |
| pH | 1 | 0.779 | 0.779 | 168.247 | 0.000 | *** | NO_3_^-^-N | 1 | 890.698 | 890.698 | 54.445 | 0.000 | *** |
|  | 13 | 0.349 | 0.027 | 5.803 | 0.000 | *** |  | 13 | 268.584 | 20.660 | 1.263 | 0.263 | ns |
|  | 13 | 0.178 | 0.014 | 2.948 | 0.002 | ** |  | 13 | 139.650 | 10.742 | 0.657 | 0.795 | ns |
|  | 56 | 0.259 | 0.005 |  |  |  |  | 56 | 916.130 | 16.359 |  |  |  |
| SOC | 1 | 144.100 | 144.100 | 23.293 | 0.000 | *** | SOM | 1 | 9808.779 | 9808.779 | 774.451 | 0.000 | *** |
|  | 13 | 44.156 | 3.397 | 0.549 | 0.883 | ns |  | 13 | 60.943 | 4.688 | 0.370 | 0.974 | ns |
|  | 13 | 47.040 | 3.618 | 0.585 | 0.856 | ns |  | 13 | 61.862 | 4.759 | 0.376 | 0.972 | ns |
|  | 56 | 346.446 | 6.187 |  |  |  |  | 56 | 709.266 | 12.665 |  |  |  |
| POC | 1 | 0.838 | 0.838 | 1.981 | 0.165 | ns |  |  |  |  |  |  |  |
|  | 13 | 14.457 | 1.112 | 2.629 | 0.006 | ** |  |  |  |  |  |  |  |
|  | 13 | 9.074 | 0.698 | 1.650 | 0.099 | ns |  |  |  |  |  |  |  |
|  | 56 | 23.685 | 0.423 |  |  |  |  |  |  |  |  |  |  |

Mean bulk density (BD), soil water content (SWC), soil pH, soil total nitrogen (TN), total phosphorus (TP), soil total potassiumsoil (TK), available phosphorus (AP), available potassium (AK), soil calcium(Ca), soil organic carbon (SOC), particulate organic carbon (POC), mineral-associated organic carbon (MAOC), total dissolved nitrogen (TDN), dissolved organic nitrogen (DON), total dissolved phosphorus (TDP), dissolved organic phosphorus (DOP), Ammonium nitrogen (NH4+–N), Nitrate nitrogen (NO3−–N), soil organic matter (SOM), Microbial biomass carbon (MBC), Microbial biomass nitrogen (MBN).

**Table S4** Two-way ANOVA results for the effects of fertilization treatment, year, and their interaction on alfalfa agronomic traits.

| Trait | Source of variation | df | Sum of Squares | Mean Square | F value | P value | Sig |
| --- | --- | --- | --- | --- | --- | --- | --- |
| Hay yield | Year | 1 | 52529396 | 52529396.000 | 175.958 | 0.000 | *** |
|  | Treatment | 13 | 97788621 | 7522202.000 | 25.197 | 0.000 | *** |
|  | Year × Treatment | 13 | 15803449 | 1215650.000 | 4.072 | 0.000 | *** |
|  | Residuals | 56 | 16717926 | 298534.000 |  |  |  |
| Heigth | Year | 1 | 3974 | 3974.000 | 74.258 | 0.00 | *** |
|  | Treatment | 13 | 4380 | 337.000 | 6.296 | 0.00 | *** |
|  | Year × Treatment | 13 | 1292 | 99.000 | 1.857 | 0.06 | ns |
|  | Residuals | 56 | 2997 | 54.000 |  |  |  |
| Stem Diameter | Year | 1 | 18.06 | 18.060 | 165.004 | 0.00 | *** |
|  | Treatment | 13 | 6.401 | 0.492 | 4.498 | 0.00 | *** |
|  | Year × Treatment | 13 | 1.357 | 0.104 | 0.954 | 0.51 | ns |
|  | Residuals | 56 | 6.129 | 0.109 |  |  |  |

**Table S5** Two-way ANOVA results for the effects of fertilization treatment, year, and their interaction on rhizosphere bacterial diversity indices.

| Trait | Source of variation | df | Sum of Squares | Mean Square | F value | P value | Sig |
| --- | --- | --- | --- | --- | --- | --- | --- |
| Sobs | Year | 1 | 211553448.048 | 211553448.048 | 1159.301 | 0.000 | *** |
|  | Treatment | 13 | 1793337.619 | 137949.048 | 0.756 | 0.701 | ns |
|  | Year × Treatment | 13 | 1914719.619 | 147286.125 | 0.807 | 0.650 | ns |
|  | Residuals | 56 | 10219080.667 | 182483.583 |  |  |  |
| Shannon | Year | 1 | 21.989 | 21.989 | 114.040 | 0.000 | *** |
|  | Treatment | 13 | 6.238 | 0.480 | 2.488 | 0.009 | ** |
|  | Year × Treatment | 13 | 3.276 | 0.252 | 1.307 | 0.237 | ns |
|  | Residuals | 56 | 10.798 | 0.193 |  |  |  |
| Simpson | Year | 1 | 0.001 | 0.001 | 8.021 | 0.006 | ** |
|  | Treatment | 13 | 0.003 | 0.000 | 2.507 | 0.009 | ** |
|  | Year × Treatment | 13 | 0.002 | 0.000 | 1.569 | 0.123 | ns |
|  | Residuals | 56 | 0.006 | 0.000 |  |  |  |
| Chao1 | Year | 1 | 283048969.324 | 283048969.324 | 1523.588 | 0.000 | *** |
|  | Treatment | 13 | 1995440.091 | 153495.392 | 0.826 | 0.631 | ns |
|  | Year × Treatment | 13 | 2195456.732 | 168881.287 | 0.909 | 0.549 | ns |
|  | Residuals | 56 | 10403560.853 | 185777.872 |  |  |  |
